# Bioremediation of clay with high oil content and biological response after restoration

**DOI:** 10.1038/s41598-021-88033-w

**Published:** 2021-05-06

**Authors:** Xiaokang Li, Jinling Li, Chengtun Qu, Tao Yu, Mingming Du

**Affiliations:** 1 College of Chemistry and Chemical Engineering, Shaanxi Oil and Gas Pollution Control and Reservoir Protection Key Laboratory, Xi’an Shiyou University, Xi’an, 710065 China; 2 State Key Laboratory of Petroleum Pollution Control, CNPC Research Institute of Safety and Environmental Technology, Beijing, 102206 China

**Keywords:** Biotechnology, Environmental sciences, Energy science and technology

## Abstract

The clay with high oil content form soil lumps, which is hard for microbes to repair. In this paper, the bioaugmentation and biostimulation technology  were applied to improve the bioremediation effect of the soil with high oil content, that modified by local cow dung and sandy soil, the ecological toxicity of the soil after restoration was further analyzed. After 53 days of bioremediation, the degradation efficiency with respect to the total petroleum hydrocarbons (TPH) content reached 76.9% ± 2.2%. The soil bacterial count of M5 group reached log10 CFU/g soil = 7.69 ± 0.03 and the results were better than other experimental groups. The relative abundances of petroleum-degrading bacteria added to M5 remained high (*Achromobacter* 9.44%, *Pseudomonas* 31.06%, and Acinetobacter 14.11%), and the proportions of some other indigenous bacteria (*Alcanivorax* and *Paenibacillus*) also increased. The toxicity of the bioremediated soil was reduced by seed germination and earthworm survival experiments.

## Introduction

During the mining, transportation, and refining of oil and gas, the leakage of amounts of oil causes lasting environmental damage. Nearly 3 billion tons of oil is exploited annually worldwide, of which 8 million tons flow into the environment, producing as much as 1 billion tons of contaminated soil and sludge. Petroleum oil bind to soil particles, reducing soil porosity and obstructing the effective absorption of nutrients by petroleum-degrading bacteria. Moreover, petroleum oil can also attach to the surfaces of plant roots and prevents plants from absorbing nutrients, leading to the death of various plants and the subsequent worsening of soil pollution^1^. As time progresses, pollutants spread from the soil to the inhabited environment, thus threatening human health and poisoning the ecological environment^2^. The longer the petroleum pollutants enter the soil, the more harmful it is to the soil ecology, which is related to the type of the soil. Clay, as a kind of soil, is sticky and barren, and hard to be degraded by microorganisms after the oil pollution, resulting in long-term existence in the soil. Therefore, physical and chemical remediation methods have been used to repair clay with high oil content, however, which may cause soil damage and secondary pollution. Based on the remediation concepts of energy savings, environmental protection, and no secondary pollution, microbial remediation technology is considered a promising method for treating  of petroleum-contaminated soil and greatly increases soil fertility and ecological diversity^3^. However, there is currently rare case of microbial remediation for high oily clay.

The soil in Xinjiang, China, is polarized, including sandy soil and clay. The soil in some oily-contaminated areas has high oil content, high viscosity, dryness and barrenness that are not conducive to the survival of microorganisms, so the soil microbial count is low. Bioaugmentation can make up for the disadvantage of insufficient microbial biomass in clay, and through adding exogenous microorganisms to the soil can improve the specific degradation efficiency of petroleum. As a method to improve microbial degradation activity in soil, biostimulation, can meet biological metabolic requirements through the addition of nutrients to soil^4^, and the recent studies have reported the efficiency of biostimulation to improved microbial degradation for petroleum oil through enhance gene allocation^5^. There are many types of stimulating substances that can improve the microbial degradation effects of soils, and different stimulating substances have varying effects on the promotion of microbial degradation activity. For example, the effect of methylene urea on the promotion of microbial degradation activity was greater than that of urea in a preferred nitrogen source^6^. Moreover, lecithin has better effects on the promotion of microbial degradation activity than certain synthetic surfactants^7^. These reported substances can be combined as biostimulants for soil bioremediation to improve the bioremediation of microorganisms and have no side effects on the soil environments.

In view of the characteristics of oil-contaminated clay, this paper proposes a simple and effective bioaugmentation and biostimulation technology, which was applied to modified petroleum-contaminated clay to reduce oil content and viscosity, and repair the oil on the clay particles. According to the local resource conditions, the present experiment used composted cow dung from a local farm and a large amount of sandy soil from the Gobi Desert as modified materials to modify clay with high oil content and used exogenous bacteria to repair modified clay. The purpose of which is to explore a simple and effective bioremediation method to repair clay with high oil content, analyze the restoration effect of microorganisms on the modified soil. The ecological restoration ability of the modified soils was evaluated to ensure the vegetation cover in the later stage.

## Materials and methods

### Preparation of modified clay with high oil content

Two types of soil samples from Karamay, Xinjiang, China (45.59 N latitude and 84.77 E longitude), including clay with high oil content from an area near oil fields and sandy soil from near the Gobi Desert, were used to prepare the experimental soil. Animal and plant residues were removed from the sandy soil, which was dried naturally and sieved to 2 mm. The clay with high oil content was ground, animal and plant residues were sieved out, and then the material was stirred thoroughly to mix the oily clay particles evenly. Cow dung from local farms in Xinjiang, China, was dried and broken into small pieces by cutting.

### Isolation of oil degrading bacteria

The isolated strains were cultured and inoculated in 1% crude oil in an inorganic salt medium for 7 days to analyze their oil degradation ability. The solution extracted with CCl_4_ from the inorganic salt medium was analyzed by gas chromatography (GC)–mass spectrometry (MS) (GC Ultra-MS 220-5880, Shenzhen Ruisheng Technology Co., Ltd., USA). The inorganic salt medium comprised 4.8 g K_2_HPO_4_·3H_2_O, 1 g (NH_4_)_2_SO_4_, 1.5 g KH_2_PO_4_, 0.2 g MgSO_4_·7H_2_O, 0.5 g Na_3_C_6_H_5_O_7_·2H_2_O, 0.002 g CaCl_2_·2H_2_O, and 1000 mL distilled water. Methylene urea was purchased from Shanghai Hengfei Biotechnology Co., Ltd., China. Tween 80 and lecithin were purchased from Beijing Houde Biotechnology Co., Ltd., China. Plant seeds were purchased from local seed companies. Earthworms were obtained from a local earthworm breeding facility.

### Treatment design and methods

#### Treatment of groups design

Oil-degrading bacteria isolated from oil-contaminated soils were cultured in a sterile lysogeny broth medium in a constant-temperature incubator shaker (180 rpm, 308 K, 24 h, and model 80501). The bacteria were purified by repeated centrifugation of the solution (9000 rpm, 277 K, 15 min, centrifuge model TG20G, Changzhou Liangyou Instrument Co., Ltd. China), decantation of the supernatant, and resuspension of the supernatant in a sterile saline solution (0.85%); this process was repeated three times. The prepared bacterial solution (OD_600_ = 0.5) was added to the soil of each experimental group.

Six experimental groups (M1–M6) were used in Table 1: M1–M5 was employed for bioremediation comparison, and M6 was the control group used for subsequent toxicity evaluation. Three parallel groups were used for each set of experiments. Nutrients and bacterial solution were added to the modified clay (385 g). Nutrient solution (inorganic salt medium, methylene urea, lecithin and Tween 80) was used to improve bacterial activity. Methylene urea was used to adjust the C:N ratio of the soil to 10:1^8^. Lecithin and Tween 80 were added to the nutrient solutions to obtain concentrations of 3 and 5 g/L, respectively. In each experimental group,  a 500 mL beaker was used as a reaction container for the contaminated soil, and the moisture content of the soil was periodically adjusted to 20–25% by adding sterile distilled water. The soil was flipped to increase its oxygen content every day.

**Table 1:** Information about the experimental groups analyzed in the present study.

Group	Composition
M1	Natural attenuation (NA): 100 g distilled water + 385 g modified clay c (45 wt% clay with high oil content, 55 wt% sandy soil)
M2	Biostimulation-1 (BS-1): 100 g nutrient solution + 385 g modified clay c (45 wt% clay with high oil content, 55 wt% sandy soil)
M3	Biostimulation-2 (BS-2): 100 g nutrient solution + 385 g modified clay d (45 wt% clay with high oil content, 45 wt% sandy soil,10 wt% cow dung)
M4	Bioaugmentation (BA): 75 g distilled water 385 g modified clay d (45 wt% clay with high oil content, 45 wt% sandy soil, 10 wt% cow dung) + 25 g bacterial solution
M5	Biostimulation (BS) + bioaugmentation (BA): 75 g nutrient solution + 385 g modified clay d (45 wt% clay with high oil content, 45 wt% sandy soil, 10 wt% cow dung) + 25 g bacterial solution
M6	Control group for toxicity evaluation:385 g natural soil (45 wt% clay + 55 wt% sandy soil)

*wt%* weight fraction, *c and d* modified clay of different proportion in Table 3.

#### Characterization of petroleum hydrocarbons, bacteria, dehydrogenase during bioremediation

During the bioremediation process of each experimental group, 10 g of soil was regularly obtained from three parallel samples to evaluate TPH using a Fourier transform infrared spectrometer (Thermo iS5, Nicolet Instrument Corporation, USA). The oil was extracted from the soils with an extraction solvent (CCl_4_) using an ultrasonic cell disrupter (JY88-IIN, NINGBO SCIENTZ BIOTECHNOLOGY CO, LTD, China), and the extraction process was repeated three times (each lasting 15 min). CCl_4_ was used as a reference solution, and the extracted liquid was diluted with CCl_4_ to 100 mL and then transferred to a 4-cm cuvette to measure the oil concentration in the soil^9^. The oil concentration was calculated using Eq. (1):

1 $${\text{Bacterial degradation efficiency}} = \frac{{{\text{C}}_{{0}} { } - {\text{ C}}_{{\text{t}}} }}{{{\text{C}}_{{0}} }}{ } \times { 100}\%$$

where *C*_*0*_ (mg/L) represents the initial petroleum concentration in the soil and *C*_*t*_. represents the petroleum concentration at reaction time *t*.

Bacterial content was determined using the flat coating method^10^. Soil dehydrogenase content was determined using a spectrophotometer (UV-2350, LinyiYingjia Scientific Instrument Co., Ltd., USA)^11^.

#### Characterization of the bacterial community structures in soils after bioremediation by metagenome extraction and high-throughput sequencing of the bacterial 16S rRNA gene

The bacterial DNA in M1-M5 samples after 53 days of repair was extracted using an AxyPrep DNA Gel Recovery Kit (Mo-Bio, Guangzhou Huajian Biological Technology Co., Ltd., USA) according to the manufacturer's instructions. The bacterial 16S rRNA gene copy numbers were quantified by a polymerase chain reaction (PCR) assay using primers 515F (5′-TCTTTCCCTACCGACGCTCTTCCGATCT-3′) and 926R (5′-GTGACTGGAGTTCCTTGGCACCCGAGAAATTCCA-3′). The library was constructed by a two-step PCR amplification method: first, a specific primer (F inner primer: 5′-TCTTTCCCTACCGACGCTCTTCCGATCT-3′, R inner primer: 5′-GAGTTCCTTGGCACCCGAGAATTCCA-specific primer-3′) was used to amplify and recover the target fragment. Then, the recovered product was used as a template for secondary PCR sequencing (F lateral primer: 5′-AATGATACGGCGACCACCGAGATCTACAC-barcode-TCTTTCCCTACACGACGCTC-3′, R lateral primer: 5′-CAAGCAGAAGACGGCATACGAGAT-barcode- GTGACTGGAGTTCCTTGGCACCCGAGA-3′). The goal of adding the adapters, sequencing primer, and barcode to both ends of the target fragment was to sequence the Illumina platform.

PCR amplification was performed in a 50-μL reaction system comprising 10 μL 5 × buffer, 1 μL (10 mM) dNTP, 1 μL F lateral primer (10 μM), 1 μL of R lateral primer (10 μM), 1 U Phusion Ultra-fidelity DNA polymerase, and 5–50 ng of a DNA template. Then, the total amount of solution was supplemented to 50 μL by adding ddH_2_O. The second amplification was performed in a 50-μL reaction system comprising 8 μL 5 × buffer, 1 μL (10 mM) dNTP, 1 μL F lateral primer (10 μM), 1 μL R lateral primer (10 μM), 1 U Phusion Ultra-fidelity DNA polymerase, and 5μL template DNA. The total amount of solution was supplemented to 40 μL using ddH_2_O.

The PCR amplification procedure was as follows. Initial denaturation was performed at 367 K for 2 min; 8 cycles were performed at 367 K for 30 s, 329 K for 30 s, 345 K for 30 s, and 345 K for 5 min, and then the temperature was maintained at 283 K. Subsequently, 3 μL of the PCR product was detected by electrophoresis on a 1.2% agarose gel and recovered by AXYGEN's AxyPrepDNA Gel Recovery Kit. After quantification by a QuantiFluor TM-ST (TBS-380, Promega, USA), the PCR products were homogenized to construct a genomic library and then sequenced using a MiSeq (Illumina, US) 250-bp paired-end platform by Novogene (Shanghai, China).

#### Toxicity evaluation of bioremediated soils

##### Seeds germination test

The surfaces of full and unbroken certified seeds (maize, wheat, ormosia, and rapeseed) were sterilized with sodium hypochlorite. Then, 10 sterilized seeds for each group were placed in different sterilized petri dishes of uniform size, followed by the addition of 60 g of treated soil. The petri-dishes were incubated at a temperature of 310 K and examined for germination inhibition every 24 h for 7 days. The petri-dishes were covered with a breathable membrane throughout 7 days to prevent moisture from evaporating quickly from the surface of the soil. The evaluation criterion of germination was visible protrusion from seed coats. Germinated seeds were counted and picked from the petri dishes at the first count on each day until there was no further germination. All experiments were performed in triplicate. Seed germination, rhizosphere length, and germination index were determined^12^.

##### Acute toxicity test in earthworms

A total of 100 g of soil from each treatment group was placed in separate brown jars. Healthy earthworms weighing approximately 220–260 mg were selected, washed with sterile water, and dried with filter paper. Ten weighed earthworms were placed in each jar to form a group. Each jar was sealed with a breathable membrane to prevent the earthworms from escaping. The surviving number of earthworms was observed after 3 and 6 days. The death of earthworms was ascertained based on their response to acupuncture. At the end of the experiment, the earthworms of each experimental group were weighed again. This process was performed in triplicate in each group^13^.

### Statistical analysis

All results were analyzed using one-way analysis of variance (ANOVA), the mean (AV ± SD) and standard deviation of three replicate experiments were obtained. The toxicity results were analyzed by Tukey’s test, and 95% confidence level analysis was performed using the statistics program SPSS; the evaluated P values were indicated by the symbols a (extremely significant difference, p < 0.01), b (significant difference, p < 0.05), and c (no significant difference, p > 0.05).

### Ethics statement

The collection of plant seed and such as material that used in this work, all comply with the relevant institutional, national, and international guidelines and legislation.

## Results and discussion

### Analysis of physicochemical properties of modified clay

**Figure 1:**
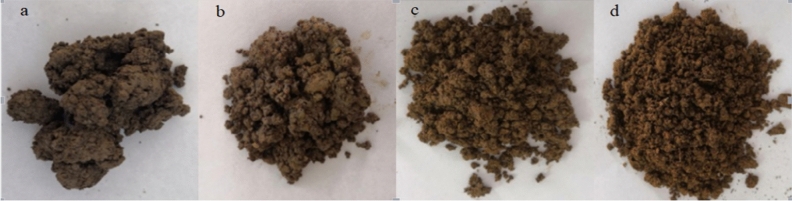
Modified clay status.

Table 2 shows the physicochemical properties of the two soils and cow dung. The clay with high oil content of 8.61 wt%, sandy soil and cow dung were mixed in a certain proportion, and then the morphologies of mixed soils with different proportions were observed. The modified clay had an oil content of 3.87 wt%. Table 3 shows the composition and proportion of different groups of modified clay, and Fig. 1 shows the status of the four groups. The soil of groups c and d had better bulkiness and contributed to the better survival of bacteria^14^. In addition, the soil viscosity of groups c and d was reduced, and the oil content of the soils was less than 6%. The toxic effect on bacteria will increase if the content of petroleum in soil is higher than 6%^15^. Therefore, the modified soils c and d were theoretically suitable for bacterial survival.

**Table 2:** Physicochemical properties of tested soil, sandy soil, and cow dung.

Parameter	Clay	Sandy soil	Cow dung
Bacterial population	3.1 × 10^4^ CFU/g	1.4 × 10^5^ CFU/g	2.3 × 10^6^ CFU/g
Organic matter	107.46 g/kg	31.75 g/kg	140 g/kg
Total phosphorus	0.69 g/kg	0.36 g/kg	1.8 g/kg
Total nitrogen	0.68 g/kg	0.73 g/kg	3.6 g/kg
pH	6.35	6.86	7.63
Moisture %	0	9.25	1.21

*CFU/g* colony forming unit per gram.

**Table 3:** Modified clay composition of four groups.

Modified clay	Modified clay composition			Soil analysis	
	Clay with high oil content %	Sandy soil %	Cow dung %	Soil oil content %	Status
a	100	0	0	8.61 ± 06	Agglomerates
b	70	25	5	6.02 ± 05	Scattered small mass
c	45	55	0	3.87	Not bonding
d	45	45	10	3.87 ± 05	Fluffy

### Identification and degradation ability of petroleum-degrading bacteria

**Figure 2:**
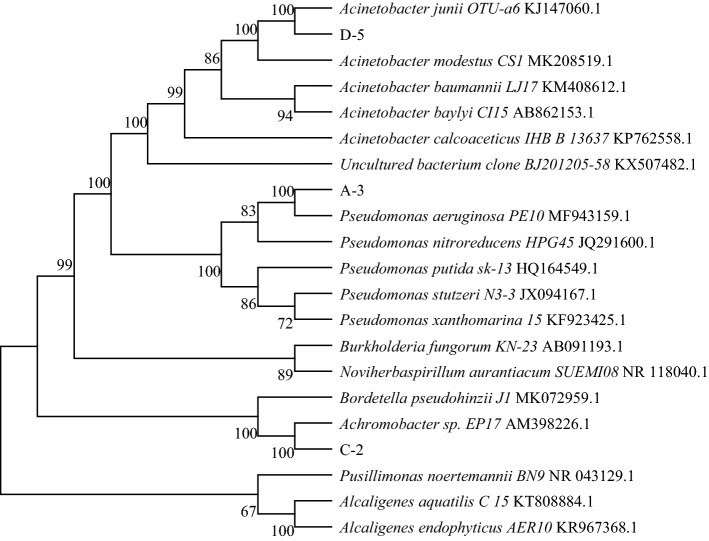
Phylogenetic tree of three test strains.

Petroleum-degrading bacteria were isolated from oil-contaminated soil near an oil field. Three kinds of petroleum-degrading bacteria (D-5 (*Acinetobacter*), A-3 (*Pseudomonas*) and C-2 (*Achromobacter*)) were identified based on 16S rRNA gene sequencing. These three bacteria belong to the phylum Proteobacteria. Figure 2 shows the phylogenetic tree of these three kinds of petroleum-degrading bacteria.

**Figure 3:**
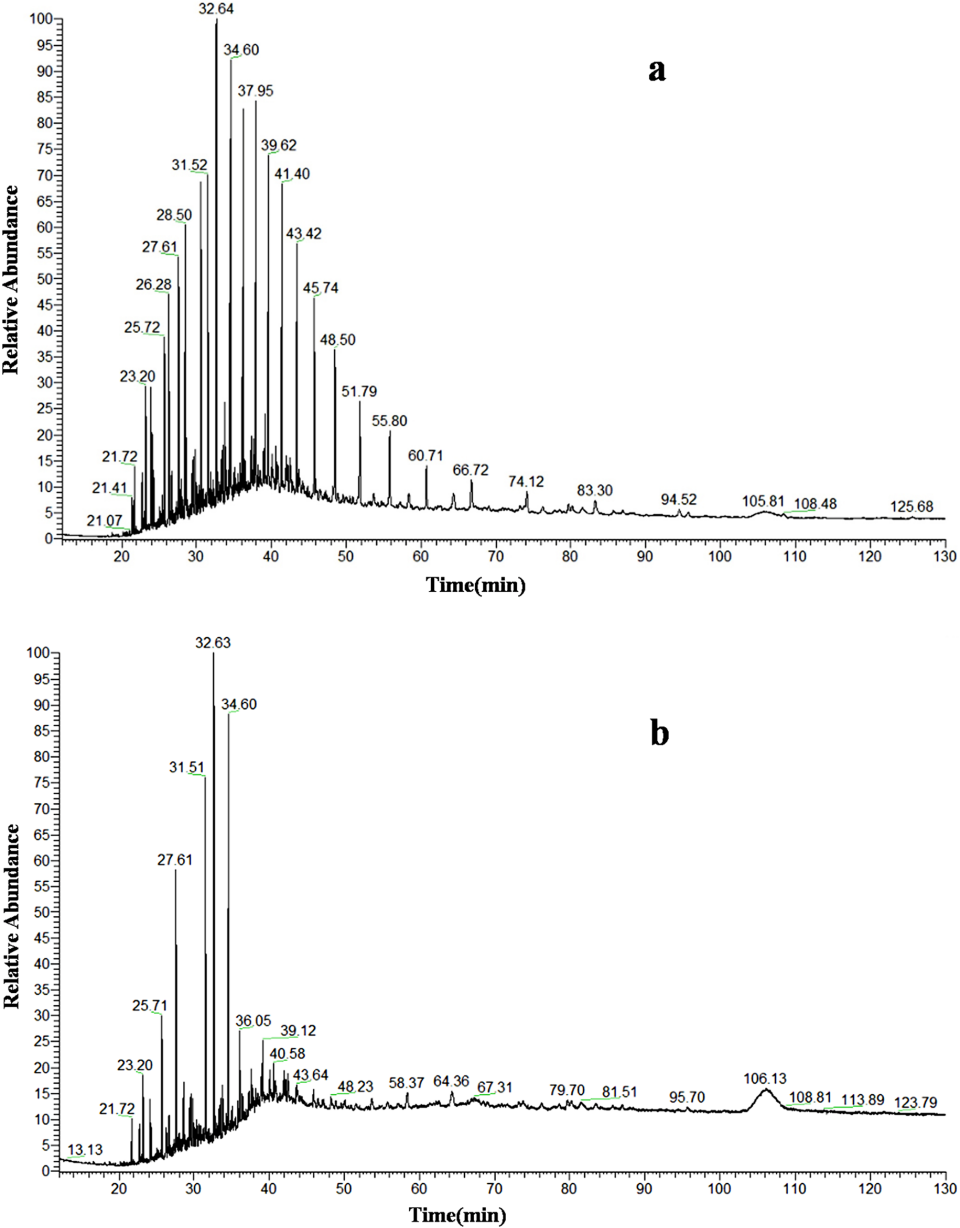
Gas chromatography mass spectrometry (GC–MS) of crude oil (**a**) before, and (**b**) after degradation.

Figure 3a shows the crude oil component before degradation, and Fig. 3b shows the crude oil component after degradation, the crude oil in the medium was degraded by the bacteria (*Acinetobacter* + *Pseudomonas* + *Achromobacter*) for 7 days. Comparing Fig. 3a with 3b shows that the bacteria degraded most of the hydrocarbons in the crude oil in a short period of time. Furthermore, the bacteria completely degraded normal paraffins in crude oil after 7 days of degradation, and some isoparaffins and other macromolecular hydrocarbons also exhibited significant degradation trends. Therefore, the screened experimental strains had good degradation effects on crude oil.

### The change of soil components during bioremediation

**Figure 4:**
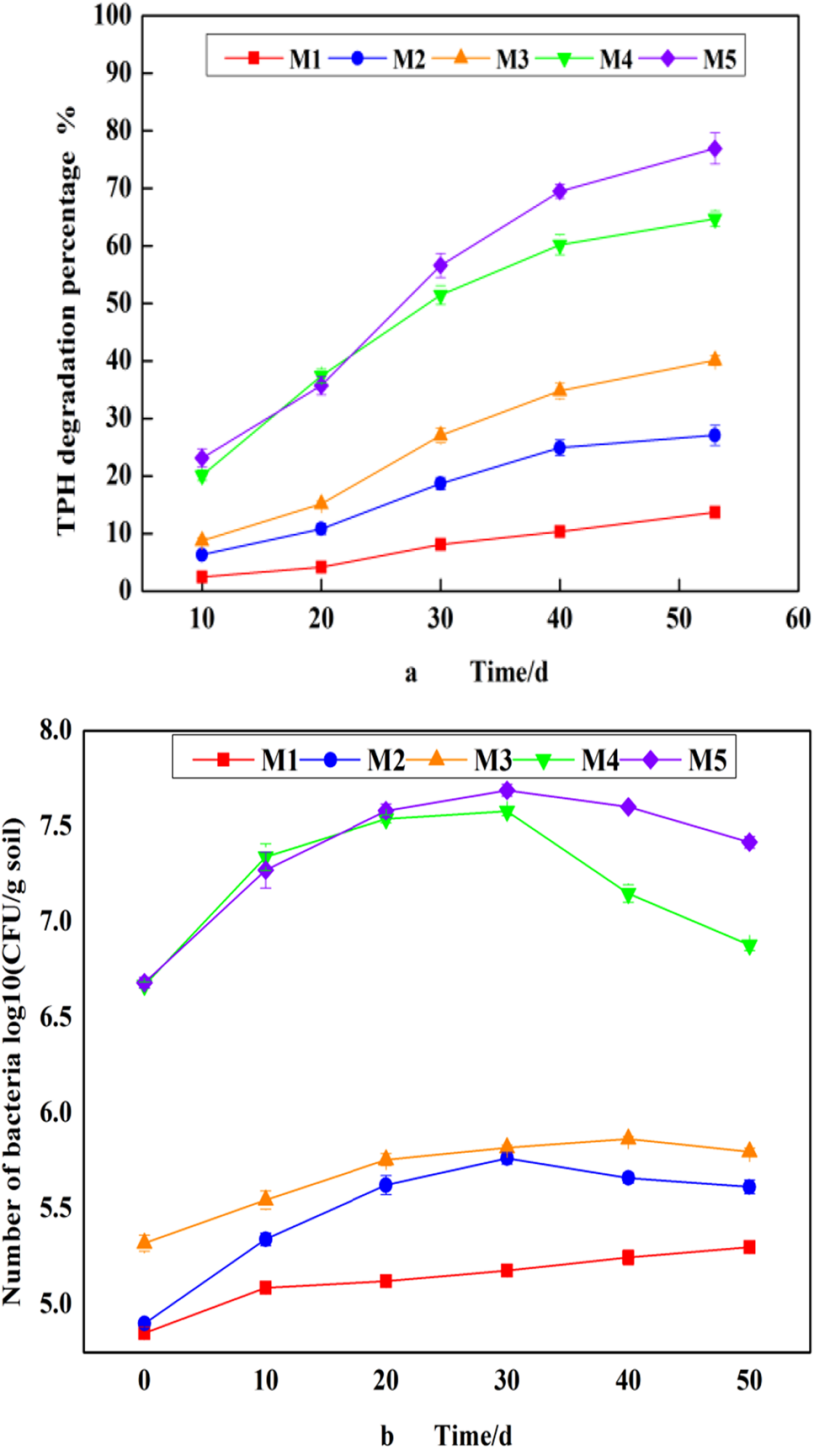
Changes in (**a**) the degradation of total petroleum hydrocarbons, (**b**) number of bacterial colonies. The results were analyzed using one-way analysis of variance to obtain the mean (AV ± SD) of three replicated experiments with standard deviation.

The crude oil degradation patterns of exogenous bacteria in M4 and M5 revealed that there was an increasing trend in crude oil degradation for each successive 10 days period up to 53 days (Fig. 4a). When the oil was degraded for 53 days, the oil degradation rate of experiment groups in soil are M1 ~ M3 < 45% (oil content > 2.1%), M4 = 64% ± 3% (oil content > 1.4%) and M5 = 76.9% ± 3% (oil content < 1%). The difference in TPH degradation activity at different remediation times was statistically significant. However, there was a slow increase in TPH degradation activity after 40 days of incubation (ANOVA, p < 0.05). It was also observed that the bacterial population in samples M4 and M5 increased from log10 CFU/g soil = 6.67 ± 0.02 to log10 CFU/g soil = 7.58 ± 0.03 and from log10 CFU/g soil = 6.68 ± 0.03 to log10 CFU/g soil = 7.69 ± 0.03, respectively (Fig. 4b). The increase in bacterial population in soils with continuous crude oil degradation implies that bacteria used various crude oil components as substrates for efficient bacterial growth. A decrease in bacterial population was observed after the 30th day of culture (Fig. 4b); For group M2, which was supplemented with nutrient solution and surfactants but no cow dung. The bacterial degradation rate was greater than that of M1, indicating that the addition of nutrient solution and surfactants (lecithin and Tween 80) effectively assisted bacterial metabolism^16^. The other surfactant (lecithin) added to the nutrient solution is an amphoteric surfactant that can produce bioelectricity to enhance the bacterial degradation efficiency. In addition, lecithin has no toxic effects on bacteria^17^.

Comparing with groups M2 and M3 (Fig. 4), cow dung was found to prolong the period of bacterial remediation, it’s maybe the cow dung provided suitable nutrients and a good living environment for the bacteria. The decrease of bacterial population in groups M3 and M5 occurred at a slow rate, which may be because the addition of cow dung and nutrients to the soil improved the activity of microorganisms. The bacterial count of the M4 experimental group decreased rapidly in the later period, which probably because no nutrient solution was added to this group. Adding only cow dung to the soil cannot effectively maintain the activity of bacteria. It is easy to cause a lack of nutrients in the later stage of degradation, resulting in a significant decrease in the number of microorganisms. Exogenous bacteria were added to the M3 (degradation rate 39.4 ± 0.4%) experimental group to obtain the M5 group, which had a significant remediation efficiency of 76.9% ± 3%. The degradation results for M3 and M5 show that the addition of cow dung and sandy soil to these groups increased the nutrient content of the soils and effectively promoted the bacterial degradation. When the mixture of cow dung and sandy soil with an appropriate proportion can significantly reduce the viscosity of clay and improve the remediation efficiency of soil. Some experiments also used the similar method to improve the degradation of soil petroleum hydrocarbons, and achieved good results. Naowasarn et al.^18^ reported that bacteria were stimulated by chicken manure to remediate oil-polluted soils and the remediation efficiency reached 60%. Agarry et al.^19^ reported that the microbial remediation efficiency was improved by adding surfactants, pig manure, and inorganic fertilizers to the soil and the remediation efficiency reached 66.4%. It shows that the nature of the soil is the basis for improving the microbial remediation efficiency of oil, and a good remediation method is also a key factor in determining the remediation effect. The modification method of oily clay can eliminate the agglomeration of oily clay during the repair process, enhance the specific surface area of the clay, and promote the contact of microorganisms with pollutants to improve the efficiency of the repair (Fig. 1). In short, nutrient (inorganic salt, methylene urea, Tween 80 and lecithin) cow dung stimulation systems can significantly improve the ability of bacteria to remediate oil pollutants in soils.

### Bacterial community structure in oil-contaminated soil after bioremediation

**Figure 5:**
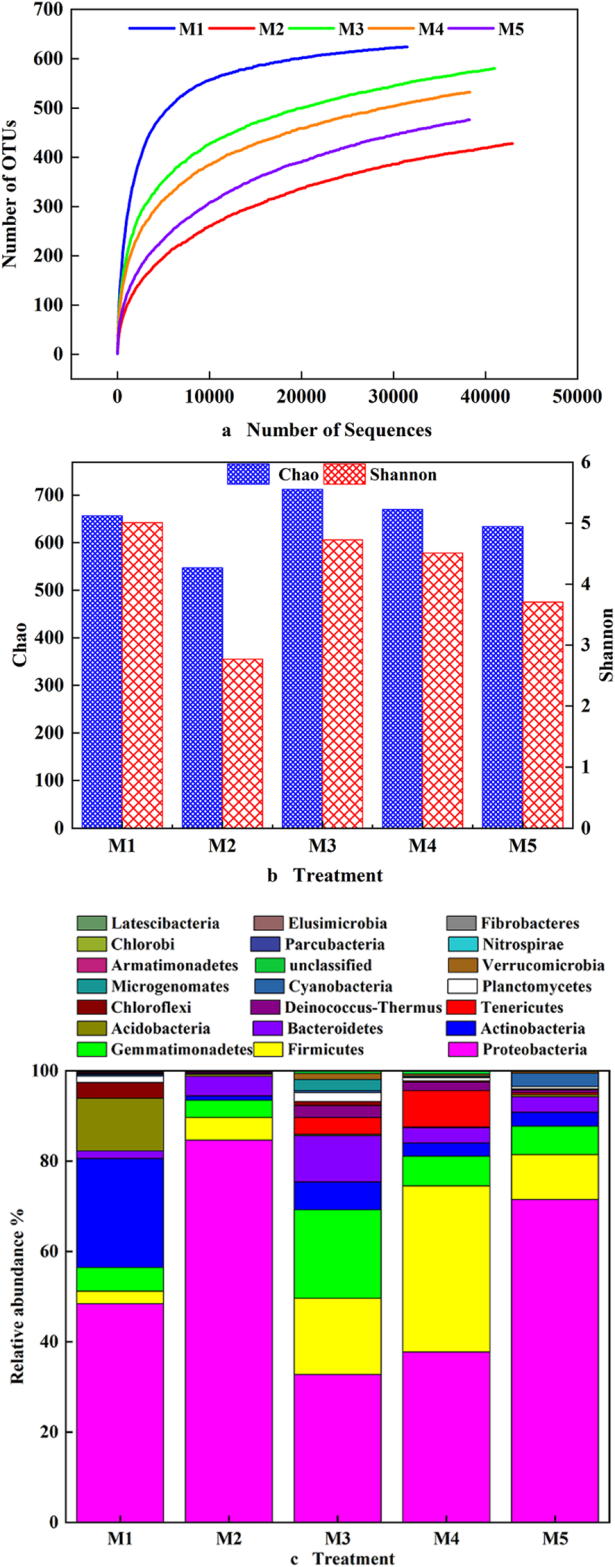
Bacterial community structures of different soil samples identified by polymerase chain reaction sequencing: (**a**) the sparse curve of the sequence obtained; (**b**) the species richness Chao index and species diversity Shannon index; (**c**) histogram of bacterial species.

A total of 225,466 sequences were obtained from the soil samples; however, only 192,036 of these were retained as optimized sequences. The number of operational taxonomic units (OTUs) in the experimental groups ranged from 428 (M2) to 624 (M1). The total number of OTUs detected in all the experimental groups reached 2640. The OTU number curves of each experimental group tended to be flat. The amount of sequencing data was reasonable, and more data would only have generated a small number of new OTUs. Therefore, the sequencing depth of the samples was obtained by making a rarefaction curve, as shown in Fig. 5a. The rarefaction curve of group M1 was completely different from those of the other groups, and 23.7% of the 2640 OTUs were unique to the modified clay samples with high oil content. The rarefaction curves of the other experimental groups indicated that the number of OTUs remained high and that there was a high level of species richness after the experiment, which would be beneficial to the natural restoration of the ecological diversity of soils. The estimated richness (Chao) and the diversity index (Shannon) were calculated from the abundance of OTUs (Fig. 5b). Compared with that of group M1, the diversity of species of the other groups decreased with the addition of nutrient solution and bacterial solutions, possibly because some bacteria in the soil could not grow and reproduce with petroleum as a carbon source or because the added bacteria competed with the indigenous bacteria in the soil, resulting in the death of some indigenous bacteria. Kong et al.^20^ reported that the addition of exogenous bacteria to an oil-containing environment increased the degradation of crude oil and altered the distribution and diversity index of bacteria. The richness and diversity of bacterial species in group M2 decreased by 16.7% and 44.6%, respectively, possibly because the addition of appropriate nutrients or surfactants could significantly change the structure of the flora and increase the abundance of petroleum-degrading bacteria^21^. Group M3 exhibited a high richness and diversity of indigenous bacteria. The bacterial diversity in group M4 did not decrease, which may be because the added bacteria had good compatibility with native bacteria, whereas the richness of bacteria changed significantly. The richness of bacterial species in group M5 remained high because of the addition of bacterial solution, whereas the species diversity decreased, which may be because the cow dung and nutrient solution were selective for bacteria in the soil.

Figure 5c shows the changes in the bacterial community structure at the phylum level. A total of 21 bacterial phyla were clearly identified after the experiments. The first three phyla were *Proteobacteria*, *Firmicutes*, and *Gemmatimonadetes*, whose relative abundance accounted for 56.49–93.68% of the total sequences. Most petroleum-degrading bacteria in these three phyla are widely used in soil bioremediation. Group M1 mainly included *Proteobacteria* (48.44%), and other phyla, such as *Actinobacteria* (24.11%), *Gemmatimonadetes* (5.29%), *Chloroflexi* (3.46%), and *Firmicutes* (2.76%), were also detected. Compared with group M1, the bacterial community structure of group M2 changed drastically at the phylum level, and a large number of sequences were assigned to *Proteobacteria* (84.67%) (Fig. 5c). The 16S rRNA gene copy number of group M1 was 6.3 × 10^6^/g soil, and that of group M2 was 7.4 × 10^6^/g soil. Therefore, the addition of nutrients and surfactant changed the bacterial community structure and relative abundance of *Proteobacteria* and *Firmicutes* in group M2. The bacterial community structure in group M3 changed partially at the phylum level. Furthermore, the relative abundances of *Actinobacteria* (6.24%), *Bacteroidetes* (10.18%), *Firmicutes* (16.85%), and *Gemmatimonadetes* (19.58%) significantly increased compared with those in group M1, and the relative abundance of *Proteobacteria* (32.79%) decreased (Fig. 5c), possibly because the addition of cow dung to the modified clay introduced new bacteria into the soil and changed the bacterial community structure, indicating that the addition of nutrients was suitable for the bacterial growth and reproduction of *Proteobacteria*, *Actinobacteria*, *Gemmatimonadetes*, *Bacteroidetes*, and *Firmicutes*. The relative abundance of *Firmicutes* (36.79%) in group M4 increased significantly compared with that in group M1. In addition, the cell membranes of bacteria of this phylum have high peptidoglycan content and are highly resistant to toxic substances, indicating that most *Firmicutes* bacteria exhibit better bacterial activity and degradation efficiency than do other bacteria in severe oil contamination conditions^22^. The relative abundance of *Proteobacteria* (71.53%) was observed to increase significantly in group M5, whereas the relative abundances of *Firmicutes* (9.92%) and *Gemmatimonadetes* (6.31%) increased compared with those in group M1. Because the bacterial population in group M5 reached log10 CFU/g soil = 7.42 ± 0.02 (Fig. 4b), the number of bacteria in *Proteobacteria* was greater. The main reason for this result was that several strains of exogenous bacteria added to the experimental groups belonged to *Proteobacteria* which increased the bacterial degradation efficiency for oil.

Table 4 shows the bacterial community composition at the genus level. At 53 days, M1 was dominated by unclassified taxa at the genus level (78.6%), and the proportion of sequences of unclassified genera in other groups was 17.8% for M2, 52.1% for M3, 34.7% for M4 and 23.1% for M5. This result suggests that the decreasing phylogenetic diversity is likely attributed to selection stress for certain genera due to the addition of nutrient solution or bacterial solution or using a different clay modification method. With the addition of nutrient solution, the majority of sequences were assigned to the genus *Alcanivorax* (17.6% in M2; 8.53% in M3; 7.27% in M4; and 12.2% in M5), which was present in the original soil before modification. This genus was previously reported to be a hydrocarbon-degrading bacterium and has been cultured and applied to various types of soils for bioremediation^23^. *Alcanivorax* had a lower relative abundance in M1 and M4 because no nutritional solution was added to M1 and M4, precluding effective stimulation of the growth of this genus; however, the cow dung added in M4 has a certain stimulation effect on the growth of *Alcanivorax*, and this stimulation effect is lower than the effect of nutrient solution on bacteria. The genera *Pusillimonas* and *Paenibacillus*, whose relative abundances improved, were previously reported to be hydrocarbon-degrading bacteria and that have been cultured and applied to various types of soils for bioremediation^24^. In M4 and M5, with added bacterial solution, the relative abundances of the genera *Pseudomonas* (12.74% in M4; 27.06% in M5), *Acinetobacter* (9.01% in M4; 14.11% in M5) and *Achromobacter* (5.05% in M4; 9.44% in M5) were significantly increased (Table 4). It may be that the addition of bacterial solution (including *Pseudomonas, Acinetobacter* and *Achromobacter*) to groups M4 and M5 caused the relative abundance of these three kinds of bacteria to maintain a higher trend in the soil. Since the genera *Pseudomonas*, *Acinetobacter* and *Achromobacter* are eutrophic bacteria, the results for M4 suggest that the modified clay stimulated the growth of bacteria, but M5, which was similar to M4 but contained added nutrient solution, had higher relative abundances of these genera. This result suggested that modified clay d (Fig. 1) can provide nutrients for petroleum degradation by bacteria and that added nutrient solution can further improve the degradation ability of bacteria.

**Table 4:** Genus level affiliation of the sequences obtained from the soil samples and the proportion of each genus in each soil sample (%).

Phylum	Class	Order	Family	Genus	M1	M2	M3	M4	M5
*Proteobacteria*	*Alphaproteobacteria*	*Caulobacterales*	*Caulobacteraceae*	*Brevundimonas*	0.01	0	0.61	1.96	0.68
				*Phenylobacterium*	1.09	1.23	1.05	2.03	0.66
			*Rhodospirillaceae*	*Ferrovibrio*	0.39	0.37	1.24	0.01	0
				*Defluviicoccus*	0.16	0	0.46	0.20	0.01
	*Unclassified Alphaproteobacteria*				4.83	2.31	2.44	3.26	1.19
	*Betaproteobacteria*	*Alcaligenaceae*	*Alcaligenaceae*	*Pusillimonas*	0.42	2.09	2.89	1.91	2.06
				***Achromobacter***	0.02	12.69	3.01	5.05	9.44
		*Comamonadaceae*	*Comamonadaceae*	*Ramlibacter*	2.16	1.21	6.02	0.03	1.06
				*Caenimonas*	0.6	0	2.05	0	0.01
			*Oxalobacteraceae*	*Herbaspirillum*	0.13	0	0	0.01	0.02
				*Massilia*	0.1	0	0.38	0	0
	Unclassified Betaproteobacteria				2.06	1.38	0.87	0.64	0.16
	*Gammaproteobacteria*	*Pseudomonadales*	*Pseudomonadaceae*	***Pseudomonas***	0.07	17.16	4.46	12.74	27.06
			*Moraxellaceae*	*Perlucidibaca*	2.28	0	0.15	0.09	0
				***Acinetobacter***	1.23	16.1	3.13	9.01	14.11
		*Xanthomonadales*	*Xanthomonadaceae*	*Arenimonas*	0.02	0	0	0.01	0
			*Pseudoxanthomonas*	*Pseudoxanthomonas*	0.13	4.42	0.01	0	0.72
		*Oceanospirillales*	*Alcanivoracaceae*	*Alcanivorax*	0.56	17.6	8.53	7.27	12.2
	Unclassified Gammaproteobacteria				3.49	1.97	1.07	1.24	2.98
*Actinobacteria*	*Actinobacteria*	*PropionibacterialesCorynebacteriales*	*Nocardioidaceae*	*Nocardioides*	5.75	0	0.12	0.04	0.03
		*Corynebacteriales*	*Nocardiaceae*	*Nocardia*	4.35	2.45	0.15	1.07	0
				*Rhodococcus*	0.58	0	0.04	2.61	1.01
	Unclassified Actinobacteria				5.24	2.31	4.15	1.12	2.5
*Firmicutes*	*Bacilli*	*Bacillales*	*Bacillaceae*	*Bacillus*	0.36	1.41	4.34	9.59	1.42
			*Paenibacillaceae*	*Ammoniphilus*	0.07	0	1.02	1.01	0
				*Paenibacillus*	0.08	2.07	0.72	4.41	1.83
				*Planomicrobium*	0.84	0.18	7.57	6.28	2.12
Unclassified Bacilli					1.42	1.14	4.23	2.32	1.05
Other					36.18	2.26	14.35	8.20	1.32
Unclassified Bacteria					25.36	9.65	24.94	17.87	16.02

### Seed germination in oil-contaminated soils after bioremediation

**Figure 6:**
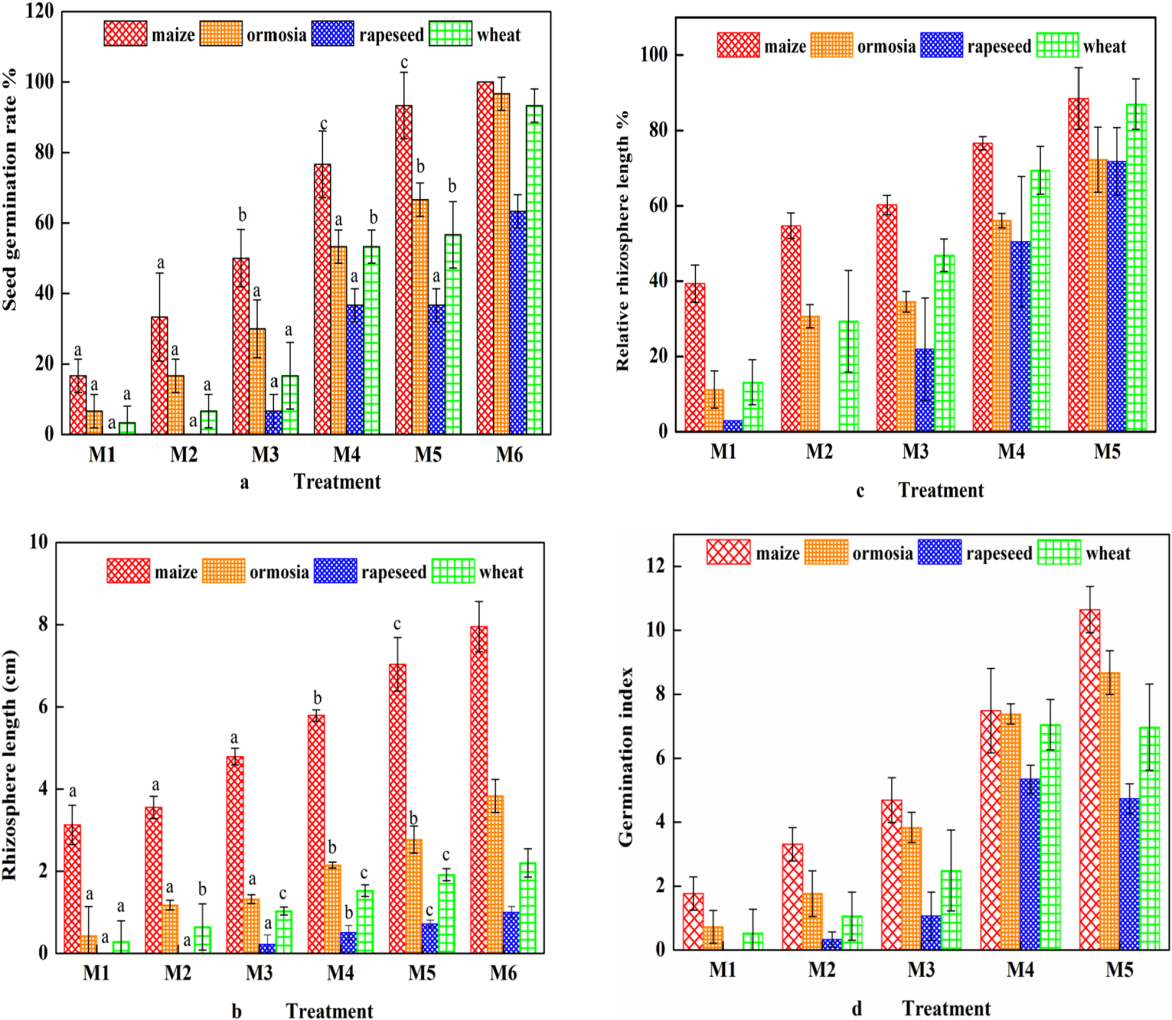
Seed germination in oil-contaminated soil after different bioremediation treatments: (**a**) the germination rate, (**b**) rhizosphere length, (**c**) relative rhizosphere length, and (**d**) germination index of different seeds. The symbols a (extremely significant difference, p < 0.01), b (significant difference, p < 0.05), and c (no significant different, p > 0.05) indicate the t-test results compared with the data for control group M6.

The growth status of plants can significantly reflect the ecological toxicity of soils. Plants need to obtain nutrients from the soil for root establishment and germination; therefore, the presence of toxic substances in soils may be reflected by alterations in seed development and physiological processes. Different types of seeds exhibit significant differences in rhizosphere length and germination time in petroleum-contaminated soil^25^. A germination experiment was used to assess the short-term toxicity effects of soil by planting different types of seeds^26^. In the present study, a germination experiment was conducted on maize, wheat, ormosia, and rapeseed seeds to evaluate petroleum contamination. The results suggest that the toxicity effect on germination and seedling growth was the highest in groups M1, M2, and M3, whereas this effect decreased in groups M4 and M5, possibly because petroleum oil creates a closed oily film layer around the seeds and thus negatively impacts germination^27,28^. Similar effects of oil contaminants on the germination rate of various commercially important plant species were also reported by Banks^29^. Petroleum oil entered the interiors of seeds, generating an adverse effect on amylase and amylophosphorylase activities, thereby reducing cells' ability to absorb starch and inhibiting the synthesis of enzymes in the cells; this process caused free oxygen radicals to be converted into toxic substances that have adverse impacts on seeds^30, 31^. Figure 6a shows the inhibition of germination in all the plants and reveals that the order of inhibition of germination was M5 < M4 < M3 < M2 < M1. The seed germination rates in groups M1, M2, and M3 were still less than 50%, indicating that the soils of these groups were not suitable for growing plants. The seed germination rates in groups M4 and M5 were much closer to that in group M6 and were not significantly different for maize in each treatment group (p > 0.05). This result indicates that maize had the strongest antipollution effect among the four plants and that the germination rates of the other three plants changed significantly (p < 0.05). Figure 6b,c shows the inhibition of rhizosphere length in all the plants and reveals that the order of inhibition was M6 < M5 < M4 < M3 < M2 < M1. This result reflected that the inhibition of rhizosphere length was the highest in groups M1, M2, and M3 but lower in groups M4 and M5. Furthermore, the seed germination index in group M5 was the highest (Fig. 6d); therefore, the toxicity of the contaminated soil significantly decreased after using the BA + BS treatment. Taken together, the findings indicate that suitable bioremediation methods effectively reduce soil toxicity to plants, which is conducive to the ecological restoration of soil in the future.

### Acute toxicity test in earthworms

Earthworms are commonly used to assess soil toxicity. The composition and physical–chemical properties of soil have an important impact on the survival of earthworms. Earthworms are also extremely sensitive to toxic substances, which can significantly change their metabolism. Therefore, earthworms were used to assess environmental risks in several experiments. Some experiments used earthworm biomass, quantity and survival rate to assess the toxicity of oil contaminant in different ecological systems^32, 33^.

**Figure 7:**
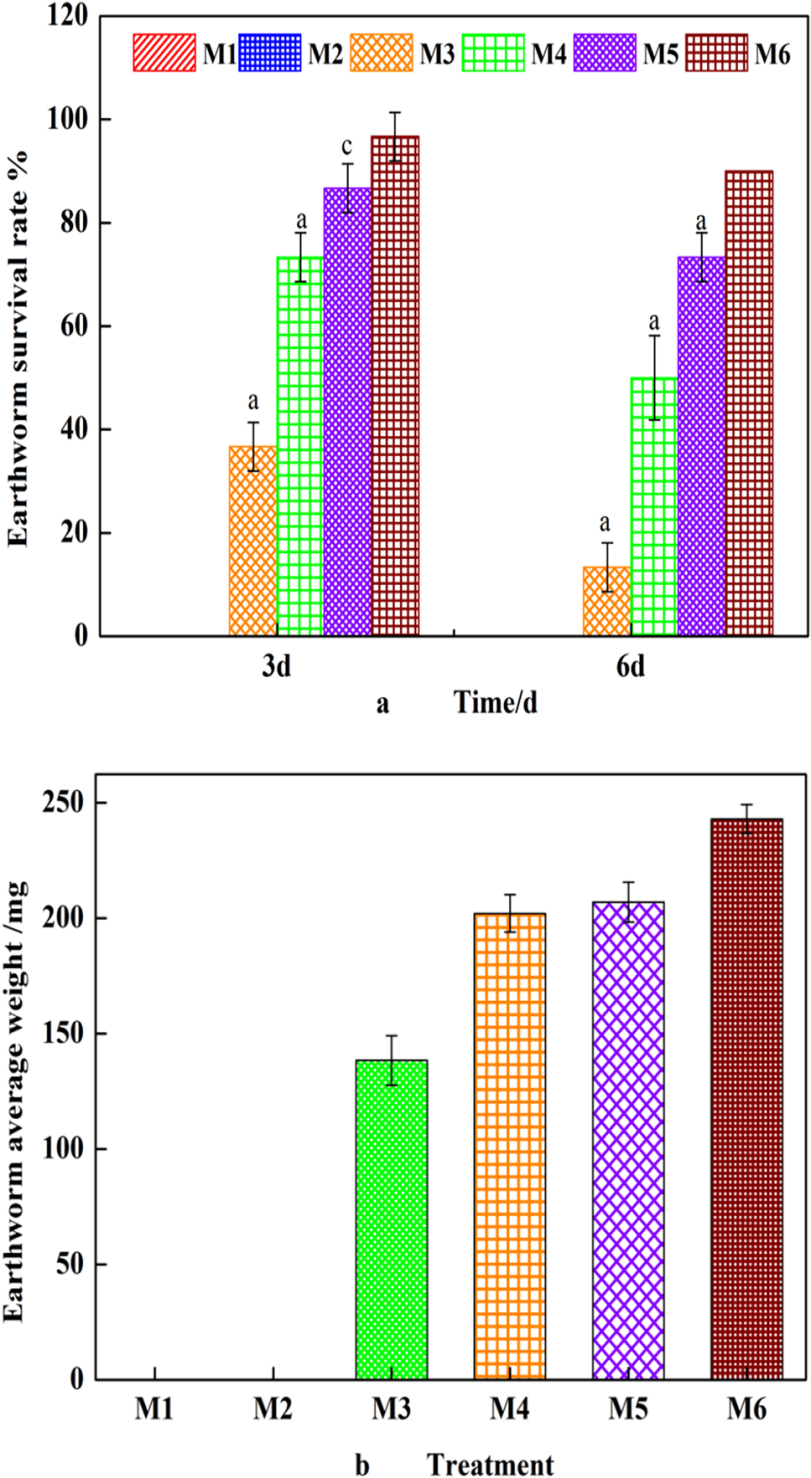
Earthworms in oil-contaminated soil after different bioremediation treatments: (**a**) survival rate and (**b**) weight of earthworms. The symbols a (extremely significant difference, p < 0.01), b (significant difference, p < 0.05) and c (no significant difference, p > 0.05) indicate the t-test results compared with the data for control group M6.

Figure 7a presents the survival rates of earthworms after 3 and 6 days in all the treatment groups. On the 3rd day of the experiment, the survival rates of earthworms in groups M1 and M2 were 0%, and that of earthworms in group M3 was less than 40%, but M4, M5 and M6 had survival rates of more than 70%. On the 6th day of the experiment, the survival rates in groups M3 and M4 were 13.6% ± 4.7% and 50% ± 8%, whereas rates of 73.3% ± 4.7% and 90% ± 0% were reported in groups M5 and M6, respectively. Figure 7b shows the weight changes of earthworms that survived in treated soil for 6 days and reveals that the weight of the earthworms was reduced in all experimental groups. The decrease in weight of earthworms in group M6 (control group) may have been influenced by the soil nutrients and living conditions. The earthworms in groups M1 and M2 rapidly moved away from the soil to adhere to the container wall 10 min after the experiment was initiated. Simultaneously, photophobism of the earthworms was not observed, indicating that the soils in groups M1 and M2 were not suitable for the survival of earthworms. During the remediation process, some earthworms adhered to a large number of soil particles, leading to a significant decrease in their life activities. Coelomic fluid was generated from dead earthworms of all the treatment groups and flowed out the body of the earthworms to adhere to a large number of soil particles. Similar results have been reported in that earthworms secrete a large quantity of mucus when they are poisoned by petroleum oil^34^. Chachina et al.^35^ found that high oil contents in soil cause death of earthworms and inhibition of body weight gain. The survival rate of the earthworms in group M5(oil content < 1%) increased significantly by 23% with respect to that in group M4(oil content > 1.4%), possibly because the clay with low oil content decreased soil toxicity to create a better environment for earthworm reproduction. Hanna et al.^36^ reported that when the oil content in soil is more than 1%, earthworm deaths will increase significantly. When the oil content in soil is less than 1%, the inhibition of earthworms is significantly weakened, and some species of earthworms can survive, which was similar to the results of this experiment.

## Conclusions

Clay with high oil content was modified by adding certain percentages of composted cow dung and sandy soil, which reduced the oil content and viscosity of the clay and effectively improved the soil environment, thereby prolonging the effective bacterial remediation time of modified clay. The prepared nutrient solution effectively enhanced the degradation effect and content of bacteria in the soil, generated species selective to the bacteria of the modified clay, and promoted the number of petroleum-degrading bacteria. The negative impact of the remediated soil on plant and earthworms was significantly reduced, which improve the survival of small animals and plants in the later bioremediation stage. Overall, this study showed that the bioremediation method used for the M5 group can be a simple, cheap and promising way of remediating clay with high oil content.
